# A novel spiral infinity reactor for continuous hydrothermal synthesis of nanoparticles

**DOI:** 10.1038/s41598-022-11141-8

**Published:** 2022-05-21

**Authors:** Arjun Kumar Pukkella, Naga Ravikumar Varma Nadimpalli, Venkataramana Runkana, Sivakumar Subramanian

**Affiliations:** TCS Research, Tata Consultancy Services, Tata Research Development and Design Centre, Pune, 411013 India

**Keywords:** Nanoscience and technology, Chemical engineering

## Abstract

Hydrothermal synthesis is an attractive route to make nanoparticles utilizing inexpensive precursors under moderate process conditions. Though it provides flexibility and robustness in controlling particle characteristics, process scale-up for continuous production is a major challenge. A novel ‘infinity-’ shaped spiral continuous flow reactor is proposed here, to exploit the large density difference between the precursor solution and supercritical water to provide rapid mixing, leading to uniform conditions for reaction kinetics and particle growth. Hydrothermal synthesis is simulated by coupling computational fluid dynamics with population balance modeling and appropriate reaction kinetics. Simulations indicate three distinct regimes of declining, recovering, and stable flow fields. These regimes are strongly dependent on the flow ratio between the precursor solution and supercritical water. The infinity reactor provides two distinct reaction environments: initial turns of the spiral which serve as a mixed flow reactor facilitating rapid mixing and uniform reaction, followed by a plug flow reactor stabilizing the particle growth. It produces particles with a relatively small mean diameter and a narrow size distribution in comparison to the conventional batch stirred tank reactor and the T-mixer.

## Introduction

Owing to their unique physicochemical properties, nanoparticles are widely utilized in diverse industries such as chemicals, energy, water, electronics and healthcare. There has been a growing interest in the large-scale production of nanoparticles with tight specifications in terms of materials and chemical composition, particle size, particle shape, dispersion and crystallinity, to make them suitable for diverse applications. Although different synthesis routes such as precipitation, sol-gel, microemulsion, spray pyrolysis, thermal decomposition, flame synthesis, and hydrothermal synthesis have been explored and demonstrated successfully on laboratory scale for various nanomaterials, commercial scale production of nanoparticles of several materials still remains a major challenge.

Recently, hydrothermal synthesis has been gaining more attention as it utilizes inexpensive precursors and moderate process conditions while providing better flexibility and robustness in the control of particle characteristics. Although supercritical water, a key reactant, at high-pressure and temperature is used, these conditions could be considered moderate when compared to processes, such as flame pyrolysis and combustion-based gas phase synthesis that operate close to the adiabatic flame temperature.

Hydrothermal synthesis is usually carried out in batch stirred tank reactors. It is limited by the slow heating rates coupled with long process times, leading to poor control of particle characteristics. The Continuous Hydrothermal Flow Synthesis (CHFS) not only addresses some of these challenges of batch operation but can pave the way for continuous and commercial scale production of nanoparticles with highly tunable particle characteristics such as size, morphology, and crystallinity^1^.

In the CHFS, pressurized Metal Salt solution (MS), typically at a temperature of 28 $^\circ$C and a pressure of 24 MPa, and Supercritical Water (SCW), typically at a temperature of 400 $^\circ$C and a pressure of 24 MPa, are introduced into the reactor separately. They are mixed rapidly in order to attain conditions that facilitate instantaneous chemical reactions for the formation of metal oxide particles. The high concentration of metal or metal oxide coupled with the low metal oxide solubility at supercritical conditions give rise to metal or metal oxide nanoparticles^2^. The mass and heat transfer dynamics in the CHFS apparatus determine the quality of mixing, Residence Time Distribution (RTD) of chemical species, reaction rates and the evolution of particle size distribution (PSD). To design a reactor that aids in the rapid and efficient mixing of two fluids that have distinctly different physical and transport properties to support the required mass or heat transfer is a great challenge.

Lester et al.^3^ proposed design a criteria for an ideal CHFS reactor or apparatus which includes: (i) instantaneous and uniform mixing of reactants, (ii) short mean residence time of the reaction mixture, (iii) minimal preheating of the MS solution before being introduced into the reactor, but rapid heating of the same as soon as it combines with supercritical water in the reactor and (iv) effective product particle transport mechanism to not only collect the particles but also to arrest further particle growth and avoid the deposition of particles on the walls of the reactor. Besides design, another important challenge is process control for ensuring process reliability and reproducibility. The method of mixing the metal salt solution with supercritical water significantly influences heat and mass transfer rates, product particle characteristics and also product consistency^4^. Several research groups have proposed different equipment designs for mixing and continuous production of nanoparticles, such as tubular reactors^5–7^, vertical nozzle reactors^3^, counter-current mixers^8^, confined jet reactors^9^, T-mixers^10,11^, cross mixers^12^ and vortex induced mixers^13^.

Among these reactors, vertical nozzle reactor^3^ appeared to have achieved reasonable success in meeting the goals for scaling-up CHFS. The technology is being commercialized by promethean^14^. In this design, the SCW and MS are introduced counter currently into the reactor. The reactor has a tube in tube design, with SCW being introduced through a central tube ending with a conical nozzle. To achieve thin film distribution, the flow is redirected upwards by the nozzle. The MS solution is introduced from the bottom of the reactor. The reactants mix in the outer tube and exit the system through an outlet located in the outer outlet.

In this work, an elegant spiral-counter spiral patented reactor is computationally explored for its potential to scale up the CHFS^15^. The uniqueness and strengths of the design shall be subsequently discussed here. Spirals have been employed in the process industries for various applications because of the interesting fluid flow fields generated due to the centrifugal forces. The early contributions from Dean^16,17^ laid the foundation for a good understanding of the secondary flows in circular cross-sectional tubes. Mashelkar and Venkatasubramanian^18^ were perhaps the first to computationally explore the convective diffusion with reaction in coiled tubes for low Dean number conditions ($N_{De}$ $\lesssim$ 20). Dean number is defined as ${\text {Re}} \sqrt{a/R_c}$, where *a* and $R_c$ refer to the radius of the tube and the radius of curvature of the spiral.

Later, Agrawal and Nigam^19^ extended the study to both Newtonian and non-Newtonian fluids for high Dean numbers ($N_{De}\lesssim 250$). It was observed that the conversion in the coiled reactor lies between the plug flow and the laminar flow reactors. Kumar et al.^20^ numerically quantified the scalar mixing between two miscible liquids for different process conditions, such as Reynolds number (Re), Schmidt number (Sc), and the number of bends for helical channels with circular cross section and juxtaposed the results with straight tubes of the same axial length. It was found that high Reynolds number flows favor good mixing between the phases in the curved pipes because of the increased influence of secondary flows. The mixing efficiency dropped in straight tubes due to the reduction in residence time as the flow rate increases. Further, Mridha and Nigam^21^ compared Coiled-Flow Inverters (CFI) with straight helical channels and observed that mixing is superior in CFIs because periodic flow inversions in the CFI helped in enhancing radial mixing of the fluid.

The heat transfer characteristics of helical channels is also superior to straight channels because of the secondary flows that act in the plane normal to the principal flow direction, augmenting the fluid mixing and heat transfer effects^22–25^. Mandal et al.^23^ experimentally showed the enhancement of heat transfer in a CFI when compared to shell and tube and plate type heat exchangers. However, the improvement in heat transfer is offset by the increase in pressure drop.

Hohmann et al.^26^ investigated flow of various solid-liquid suspensions in helical coiled tubes experimentally and identified various flow regimes, from suspension to sedimentation. Pukkella et al.^27^ studied the effects of various process and operational parameters on the preferential classification of particles of top size ($d_p$) less than $300\mu$, in several non-circular cross-sectional spiral channels. Granados-Miralles et al.^28^ carried out experiments in a batch spiral reactor to produce ${\text {SrFe}}_{12}{\text {O}}_{19}$ nanoparticles. A dual-stage spiral reactor, resembling the shape of a spiral condenser, was proposed for the continuous production of nanoparticles^29^.

It can be noted from the brief review above that spiral reactors were not explored for nanoparticle synthesis. In the current work, a novel spiral-counter spiral reactor is proposed^15^, which takes advantage of the difference in density between SCW and MS solution. In this design, a spiral turn of clockwise flow direction is followed by an anti-clockwise spiral turn, changing the flow direction and the centrifugal force fields. It is hypothesized that this alternating force field will spur intense mixing between the reactants, especially when the density difference is significant.

**Figure 1:**
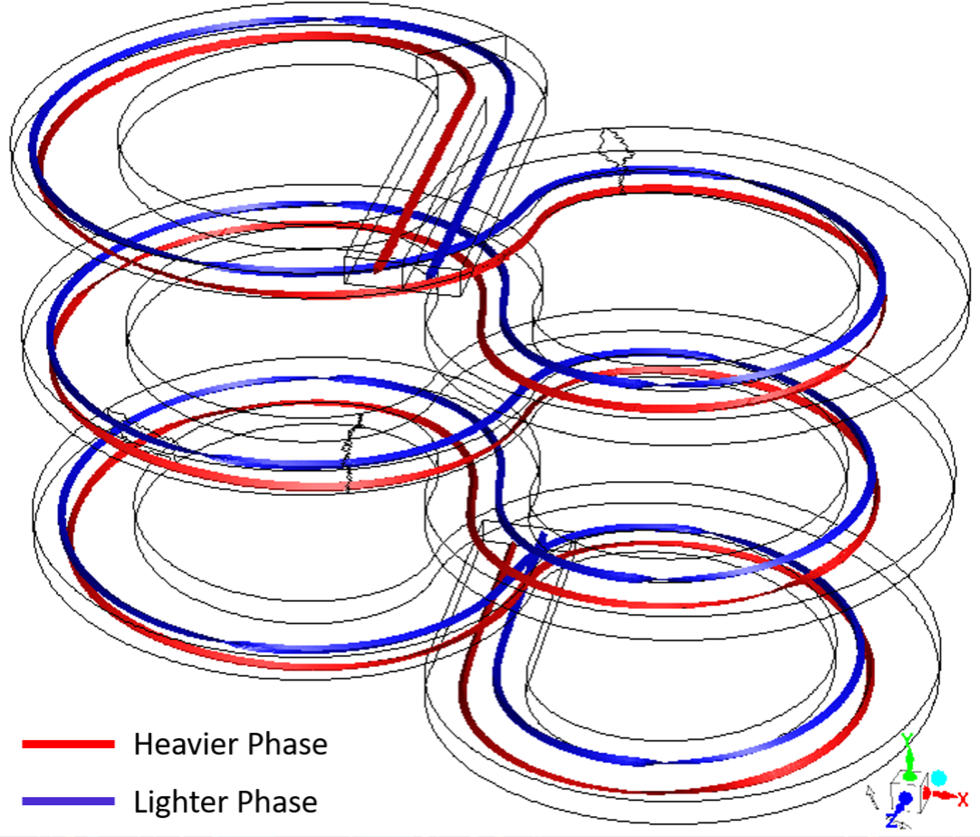
Schematic depicting the periodic cross-overs in the infinity reactor when two fluids of different densities are fed. Images used courtesy of ANSYS, Inc.

A schematic representation of how the flow paths of the two reactant streams would cross is shown in Fig. 1. Here, MS (red) and SCW (blue) streams are injected into the reactor from their respective inlet positions. The denser phase is introduced near the inner side of the spiral turn and the lighter phase is introduced towards the outer side of the turn; this is the reverse of their equilibrium positions. Due to the centrifugal force field, the heavier phase moves towards the outer side and mixes intensely with the lighter phase. When the turn is reversed, the flow dynamics is repeated, with the heavier phase interpenetrating the lighter phase, creating an environment for vigorous mixing. The reactor is christened as **infinity reactor** as its top view resembles the shape of the symbol $\infty$.

The periodic change in the flow direction is expected to create flow fields that aid rapid mixing without back mixing and stagnation zones, while maintaining the uniform residence time that is conducive for particle formation and growth. The objective of the current work is to evaluate the infinity reactor for the production of nanoparticles with good control on particle characteristics. A one-way coupled Computational Fluid Dynamics (CFD) and Population Balance Model (PBM) is employed to study the performance of the infinity reactor at different operating conditions. Based on these simulations, design and optimization criteria are proposed for better control of nanoparticle characteristics.

The rest of the paper is organized in the following manner: The mathematical modeling aspects of hydrothermal synthesis of nanoparticles are presented in section “Computational methodology”. The model implementation details including discretization of the reactor geometry and initial and boundary conditions are discussed in section “Model implementation”. Numerical simulation results are presented and discussed in section “Results and discussion” to assess the scale-up of flow rates to produce nanoparticles.

## Computational methodology

The hydrothermal synthesis of nanoparticles involves complex and coupled phenomena such as hydrodynamics, heat and mass transport, chemical reactions, particle formation and growth. Hence, integrating mathematical models representing individual phenomenon is important. In the present work, the material domain consists of the fluid and the population of nanoparticles, considered as continuous and dispersed phases, respectively. The material domain is treated using the Eulerian framework. The population of nanoparticles is treated as a separate continuum phase owing to its effect on the momentum exchange with the fluid and on the overall turbulence intensity. The criteria for the population of nanoparticle to be assumed as continuum can be found elsewhere^30^. As equations of the models are available elsewhere^30,31^, albeit for a different reactor geometry, only the essential details are recapitulated here. Synthesis of ceria nanoparticles is utilized as an example here because of their commercial importance in many different applications such as polishing material in semiconductors, photocatalyst in water treatment, fuel cell catalysts, optical films, gas sensors, redox (oxygen storage) promoters in three-way catalysts and oxygen ion conductor in solid oxide fuel cells.

### Reactions and species transport

The thermo-physical properties of the solvent such as density, dielectric constant, viscosity, thermal conductivity, and heat capacity significantly change as the reaction temperature approaches the critical point of the solvent. These properties not only control the transport phenomena of the reactants but also the chemical reactions, particle nucleation and growth steps. For example, the dielectric constant of water is approximately 78 at room temperature and this value falls steeply to 8, beyond the critical temperature. In supercritical hydrothermal synthesis, the solubility of metal oxides exhibits a unique behavior around the critical point; the solubility first increases with temperature and then decreases because of the drop in density and dielectric constant^32^. The reaction rate constant increases two-orders in magnitude when temperatures go beyond the critical point^33^. When the aqueous metal-salt solution is rapidly heated to the supercritical state, instantaneous hydrothermal reactions take place to form metal oxides because solubility of the metal oxide in this state is very low. Consequently, an extreme degree of supersaturation is attained, leading to the formation of nanoparticles.

The reactions involved in the hydrothermal synthesis of ceria nanoparticles with cerium nitrate as the precursor are:

1 $$\begin{aligned} &{\text {Hydrolysis:}}\quad 2{\text {Ce}}({\text {NO}}_{3})_{3} + 8{\text {H}}_{2}{\text {O}} \rightarrow 2{\text {Ce}}({\text {OH}})_{4} + 6{\text {HNO}}_{3} + {\text {H}}_{2} \end{aligned}$$

2 $$\begin{aligned}&{\text {Condensation:}}\quad {\text {Ce}}({\text {OH}})_{4} \rightarrow {\text {CeO}}_{2} + 2{\text {H}}_{2}{\text {O}} \end{aligned}$$

In the temperature range considered here, the hydrolysis reaction can be assumed to be instantaneous and thus, the overall reaction is represented as:

3 $$\begin{aligned} 2{\text {Ce}}({\text {NO}}_{3})_{3} + 4{\text {H}}_{2}{\text {O}} \rightarrow 2{\text {CeO}}_{2} + 6{\text {HNO}}_{3} + {\text {H}}_{2} \end{aligned}$$

The Bohr equation is used to represent the reaction rate constant ($k_r$) of the net hydrothermal reaction as a function of temperature and dielectric constant, as given below^33^:

4 $$\begin{aligned} \ln k_r = \ln k_0 -\frac{E}{RT} + \frac{\psi }{RT} \left( \frac{1}{\epsilon } - \frac{1}{\epsilon _0}\right) \end{aligned}$$

where *R*, $k_0$, and $\psi$ are the universal gas constant, frequency factor for the reaction, and a constant that depends on the reaction system, respectively; $\epsilon _0$ and $\epsilon$ are dielectric constants of the solvent at the reference temperature and system temperature, respectively. For the ceria nanoparticle system, based on their experiments in a T-mixer, Aoki et al.^34^ calculated the values of frequency factor to be $k_o = 2.8 \times 10^7\, {\mathrm{s}}^{-1}$ and parameter $\psi = 4.5\times 10^2\,{\text {kJ/mol}}$. It was reported that the Reynolds number has a negligible effect on the rate of reaction and the overall reaction obeys first order kinetics with respect to cerium nitrate concentration. As the temperature range of interest here is 472K to 613K, which is within the limits reported in the experimental study, these values are used in this work^30^. The solubility of ceria as a function of temperature (*T*) at a pH of 10 and pressure of 25 bar is captured by the following equation:

5 $$\begin{aligned} y = 0.00001823 + 1.522 \times 10^{-7}T - 4.568 \times 10^{-10}T^2 + 6.125 \times 10^{-13}T^{3} + 3.0568 \times 10^{-16}T^4 \end{aligned}$$

where *y* is solubility of ceria in [mol/kg] and *T* is reaction mixture temperature in [K]. It was developed by fitting the solubility data obtained by thermodynamic calculations^35^. This thermodynamics driven methodology was used to analyze the stability of ceria phases and maximize the yield of ceria nanoparticles in a batch hydrothermal reactor^31^.

The species transport equations along with homogeneous volumetric (bulk) chemical reaction (Eq. 3) in the reaction mixture (primary phase) is considered in the model^36^. A finite rate eddy dissipation model has been used for accounting the turbulence-chemistry interaction^37^. The species transport equations are solved for $n-1$ components, and for the most abundant *n*th component, water in this case, the mole or mass fraction is calculated by subtracting the sum of all the other mole or mass fractions from unity.

It is worth adding that in this process, ${\text {H}}_2$ formed remains in the gas phase as the conditions are well above its critical point. Its properties were computed during the simulation accordingly. Further, stability of nitric acid under the conditions considered here was also ascertained. To dissociate into nitrogen-based compounds, ${\text {HNO}}_3$ concentration should be greater than 90 weight percent at the temperatures and pressure used here^38^. In the current simulations, the weight percentage of precursor, ${\text {Ce}}({\text {NO}}_3)_{3}$ which is the source of nitric acid formation is only about 3.5 weight percent. As a result, the nitric acid formed is less than 1 weight percent. Therefore, decomposition of nitric acid is assumed to be negligible under the reaction conditions.

### Fluid flow

The high-fidelity computational fluid dynamics simulations are employed to capture the phenomena of nanoparticles synthesis in the infinity reactor. It has been reported that the multiphase Eulerian-Eulerian framework accurately captures the transport properties when compared to the mixture multiphase and single phase models^39,40^. Hence, the multiphase Eulerian-Eulerian framework is used to model the flow dynamics. In the Eulerian modeling approach, each phase is treated as continuum. The turbulent dispersion forces are also considered in the momentum equation. The rotational and lift forces on the particles are neglected because of the small size of the particles.

The Knudsen number (Kn) is calculated to be 0.006 for nanoparticles of diameter 50 nm. The recommended criterion for a phase to be treated as continuum, ${\text {Kn}} < 0.1$ is well met, Hence, the nano-particulate phase is treated as a continuum^39^. The reaction mixture consists of ${\text {HNO}}_{3}$, ${\text {CeO}}_{2}$, ${\text {Ce}}({\text {NO}}_{3})_{3}$, ${\text {H}}_{2}$, and ${\text {H}}_2{\text {O}}$ involved in the reaction (3). The reaction mixture is modeled as the primary phase whereas the ceria nanoparticles are considered to be a part of the secondary phase which exchanges momentum with the primary phase^39^. Enthalpy equation is enabled for each phase to account for the heat transfer among the phases. Heat of chemical reaction, compression or expansion of the fluid, viscous dissipation due to internal friction, heat flux, and the interphase heat transfer between the primary and secondary phases are considered in the modeling.

The two-equation realizable $k-\epsilon$ model is used to model turbulence. This model is computationally less expensive, reasonably accurate and it has been verified extensively for buoyancy driven turbulent flows^41^.

### Nucleation and growth of nanoparticles

The one dimensional population balance model given in Eq. (6) taking into account nucleation, diffusional growth and aggregation of the particles for a continuous system is employed^30^. Here, the dispersed phase consists of the population of particles that evolve. The particle volume is considered as the internal coordinate. It is a strong function of continuous variables such as fluid velocity, temperature and species concentration. Axial averages of these variables obtained from the CFD model are fed to the PBM. The diffusional growth and aggregation terms in PBM were discretized using the finite difference formulations^42,43^.

6 $$\begin{aligned} \frac{\partial }{\partial t} \left( \rho _s \alpha ^{i}_s \right) + \nabla .(\rho _s v_p \alpha ^{i}_s) + \frac{\partial }{\partial V}\left( \frac{G_V \rho _s \alpha ^{i}_s}{V}\right) = \rho _s V_i (B_{ag,i}-D_{ag,i}) + S^i \rho _s V_0 {\dot{n}}_0 \end{aligned}$$

where $\rho _s$: density of the secondary phase, $\alpha ^{i}_s$: volume fraction of the secondary phase of size class ‘*i*’, $v_p$: the primary phase velocity, $V_i$ and $V_0$: the volume of the particle of size class ‘*i*’ and the smallest particle size considered in the system, respectively, ${\dot{n}}_0$: the nucleation rate, and $G_V$: the diffusion controlled growth rate. The birth and death rates of the particles through coagulation, $B_{ag,i}$ and $D_{ag,i}$, respectively are expressed using the following equations:

7 $$\begin{aligned} B_{ag,i} = \sum _{k=1}^{N} \sum _{j=1}^{N} \beta (V_k,V_j) N_k N_j \zeta _{kj} \end{aligned}$$

with

8 $$\begin{aligned} \zeta _{kj}= & {} {\left\{ \begin{array}{ll} 1 \quad \text {for} \quad V_i< V_{ag} < V_{i+1}, &{}\quad \text {if } i\le N-1\\ 0, &{}\quad \text {otherwise} \end{array}\right. }\nonumber \\ D_{ag,i}= & {} \sum _{j=1}^{N}\beta (V_i,V_j)N_i N_j \end{aligned}$$

where $\beta (V_i,V_j)$ is the coagulation kernel.

As there are no seed particles involved, it is assumed that particles undergo homogeneous nucleation, which is well described using the classical nucleation theory. The nucleation rate is a strong function of the degree of super-saturation of the species ($\lambda$) and the interfacial energy of the ceria-water ($\sigma$) system as shown in the equation:

9 $$\begin{aligned} \dot{n_0} = A \exp { \left( \frac{-16\pi \sigma ^3 {v_m}^3}{3(k_BT)^3(\ln \lambda )^2}\right) } \end{aligned}$$

where $\lambda$: ratio of the concentration (*C*) and solubility ($C_s$) of ceria, $v_m$: volume of the ceria molecule, and $k_B$: Boltzmann constant. Further the effect of temperature on the solubility of species and the interfacial energy is expressed by the following equation:

10 $$\begin{aligned} \frac{a^2\sigma }{k_B T} = 2.82 - 0.272 \ln C_s \end{aligned}$$

In the above equation $C_s$ is expressed in ${\text {mol/m}}^3$. The diffusional growth is also controlled predominantly by super-saturation and diffusion of ceria. The interplay between transportation of ceria molecules and incorporation of ceria molecules into the existing particles determines the diffusional growth rate as given by the following equation:

11 $$\begin{aligned} G_V = \frac{dV}{dt} = 4 \pi v_m N_A DC\left( \frac{3V_i}{4\pi }\right) ^{1/3} \end{aligned}$$

where *D* is diffusion coefficient of the ceria.

The growth due to aggregation of particles because of Brownian motion and shear rate of fluid depends on particle size, temperature and collision frequency between particles^30^. The following expression captures the growth through coagulation because of Brownian motion:

12 $$\begin{aligned} \beta _{Brown}(V_i,V_j) = \left( \frac{2k_B T}{3\mu }\right) \left( \frac{1}{V_i^{1/3}}+\frac{1}{V_j^{1/3}}\right) ({{V_i^{1/3}}+V_j^{1/3}}) \end{aligned}$$

where $\mu$ is viscosity of the water.

The fluid shear induced collisions depend strongly on the particle size. The collision rate due to fluid shear is given by the Smoluchowski equation.

13 $$\begin{aligned} \beta _{shear}(V_i,V_j) = \frac{1}{\pi }(V_i^{\frac{1}{3}}+V_j^{\frac{1}{3}})^3 \frac{d\bar{v}}{dx} \end{aligned}$$

where, $d\bar{v}/dx$ is shear rate. The total coagulation frequency is obtained as the sum of the collisions due to Brownian motion and shear induced coagulation. More details on the model equations and physical parameters for the PBM implementation can be found elsewhere^30^.

## Model implementation

The unsteady flow, heat and mass transport simulations were performed using a finite volume based commercial CFD software ANSYS Fluent 19.1^36^. In this section, the design details of the infinity reactor are presented. The geometric details and the boundary conditions adopted in modeling the process are discussed first, followed by the grid independence test to select the mesh with appropriate cell density that captures the physics with the least computational cost.

### Geometry and boundary conditions

**Figure 2:**
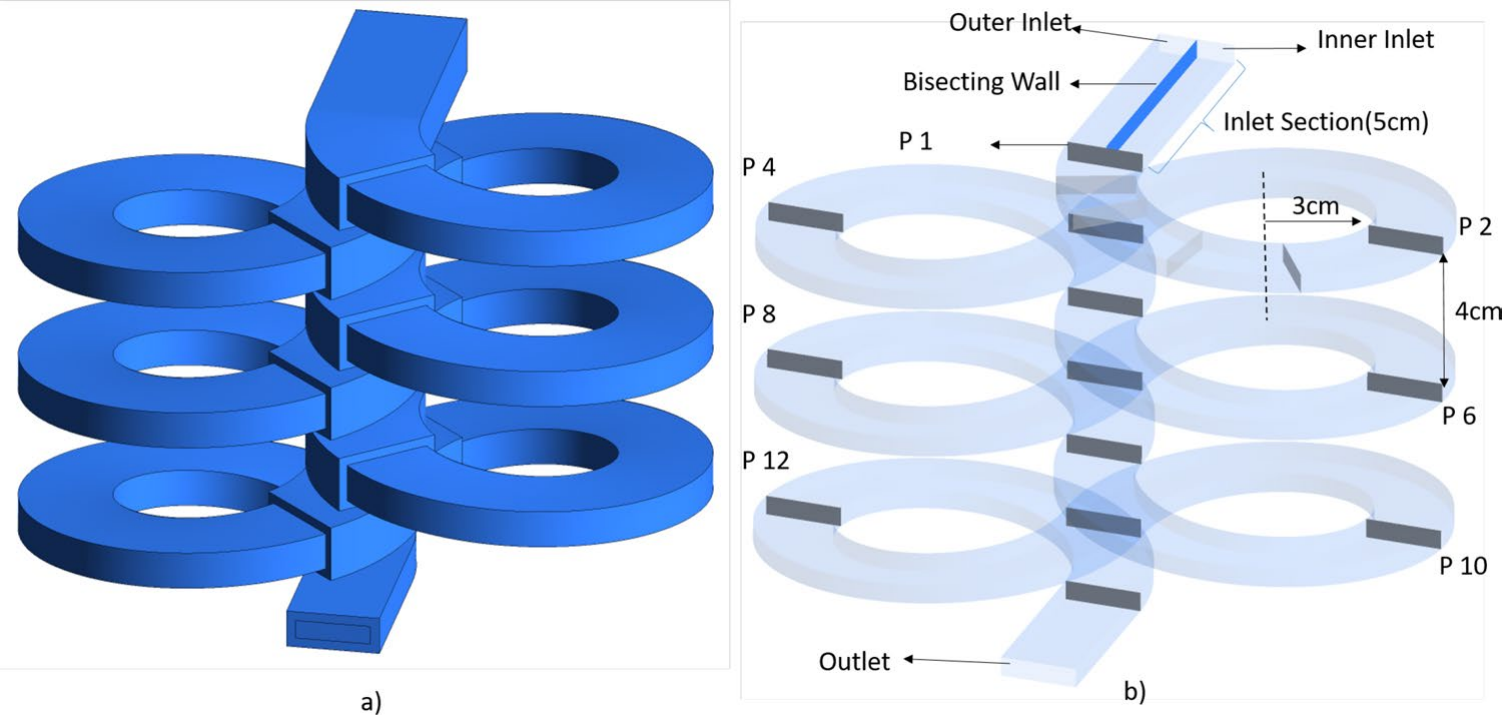
Geometry of the infinity reactor: (**a**) outside view showing the modular design, and (**b**) flow path highlighting the axial planes used for post-processing. Images used courtesy of ANSYS, Inc.

The complete geometry of the infinity reactor is shown in Fig. 2a. Figure 2b represents the flow path of the fluid domain. The infinity reactor can be designed in a modular fashion, where the number of infinity spiral sets can be added with the help of connectors or couplings, as per the process requirements. It is worth noting that for high-pressure applications such as the one under consideration here, a single monolithic construction with multiple turns might be preferred to a modular design for improved safety. Our focus is primarily on the operating aspects of the reactor. Mechanical components that are required to fabricate the system are not dealt with here.

The reactor design comprises of the inlet section that is 5 cm long wherein the metallic precursor solution and the supercritical water enter separately on the two sides of a wall placed in the inlet section, to prevent fluids from mixing. At the end of the inlet section, the mixing/reaction section starts and continues into a series of spiral channels that periodically change the flow direction. Finally, the product stream exits from the outlet section through the outlet face as shown in the figure. The grey planes (P1 to P13) located along the flow path axially in the Fig. 2b represent the pseudo planes created to perform post-processing. These planes are created for every half turn of the spiral; between planes P1 to P2 four additional planes P1a, P1b, P1c, and P1d are created to capture the rapid f mixing between the phases.

The entire flow path has a uniform rectangular cross-section with a width of 2 cm and a height of 0.5 cm. The spiral turns have a pitch, vertical distance between the centers of spiral and its counter spiral turn, of 2 cm and an inner radius of curvature of 3 cm. The thickness of the solid wall is taken as 2.5 mm for the entire reactor. Note that the simulation results obtained here shall not be sensitive to the wall thickness as the wall is taken to be insulated. The wall thickness will be an important parameter that has to be calculated rigorously taking into consideration many factors including the strength of the material used, the pressure requirements and the desired factor of safety, while fabricating the device.

Simulations were performed for various flow rates of metallic salt solution and supercritical water. The aqueous metallic precursor solution of cerium nitrate with 0.035 mass fraction enters at a temperature and pressure of 300 K, and 25 MPa, respectively. The supercritical water enters at a temperature and pressure of 673 K and 25 MPa, respectively. The system was specified to work under a constant pressure of 25 MPa. The thermo-physical properties of water, namely, density, viscosity, specific heat capacity and thermal conductivity are dependent on temperature. They were modeled using higher order polynomials and implemented through User Defined Functions (UDF) in Ansys Fluent^30^. All the inner walls in contact with the fluid were given no-slip boundary condition and treated as a coupled wall with zero heat generation rate. The outer wall was treated as a completely insulated wall with zero heat flux. The outlet was modeled as an outlet-vent boundary. A steady state solver with phase coupled Semi-Implicit Method for Pressure Linked Equations (SIMPLE) algorithm was used for pressure-velocity coupling. First-order upwind scheme was used for spatial discretization of momentum, energy, volume fraction, species transport and turbulence terms. The iterations were continued till the residuals of the governing equations reached the order of $10^{-3}$. The discretized PBM equations were solved using the Runge-Kutta implicit iterative method using the open source software Scilab^44^. The evolution of PSD was captured using 40 size classes with geometric grid discretization of particle size range 1–50 nm^43^.

### Mesh and grid independency

Considering the complexity of the geometry, an unstructured tetrahedral mesh was created in Ansys ICEM CFD software. The mesh was refined towards the solid walls in the flow domain to capture the effect of wall accurately. Coarser cells were considered for solid walls.

**Figure 3:**
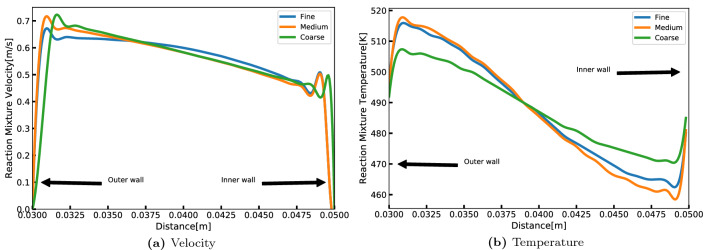
Grid independence results of centerline profiles at the end of the first spiral turn: (**a**) velocity, and (**b**) temperature.

Three sets of meshes with different cell counts were created as shown in Table 1. A representative simulation result showing the center-line radial profiles of reaction mixture velocity and temperature are plotted in Fig. 3 for the three meshes at the first half turn (Plane-2). It can be observed from Fig. 3a that velocity values are high near the outer wall region because of the compounded effect of induced centrifugal force on the reaction mixture and the fact that supercritical water is introduced from the outer-inlet with relatively much higher velocity than the metallic salt solution. It can be noticed from Fig. 3b that the temperature of the reaction mixture gradually decreases from outer wall side to the inner wall side. In the region near the wall, one can observe the reverse trend in the profiles. This is because heat capacity of the wall material, acts as a sink on the outer side and source on the inner side. In other words, the inner wall temperature on the outer side is less than the local reaction mixture temperature and vice-versa. From the predictions presented in Fig. 3 and Table 1, mesh M2 is selected to be sufficient to capture the physics without being overly conservative (high computational cost).

**Table 1:** Comparative results of tested computational grids.

Mesh	Description	$V_{max}$ (m/s)	$T_{max}$ (K)	Number of cells
M1	Fine	0.641	515	3620546
M2	Medium	0.665	517	1385987
M3	Coarse	0.695	507	496035

## Results and discussion

Simulation results of ceria nanoparticle synthesis obtained using the coupled CFD-PBM are presented in this section. As mixing and chemical reactions depend on both the absolute and relative flow rates of the two reactant streams, they are varied in the simulations, and their effect on hydrodynamics, mass transport, heat transport and particle dynamics are characterized. The relative flow is captured using the Flow Ratio (FR), defined as the ratio of the mass flow rates of MS to SCW. The flow rate of SCW was fixed at three levels, namely 1200, 2400, and 4800 g/min; simulations with a fixed SCW flow rate are referred to as a Flow Set (FS) in the article. The FR was changed from 0.25 to 1.5 in steps of 0.25 for each of these flow sets. All the simulation conditions are listed in Table 2. It is worth noting that, for the cases studied here, if 100 percent conversion is achieved, the reactor could produce ceria at the rate of 0.33–8 kg/h depending on the flow rates of MS and SCW.

**Table 2:** Simulated feed flow rate conditions, Reynolds (Re) and Richardson (Ri) numbers calculated for the reaction mixture at the end of the inlet section, predicted threshold velocity and ceria yield.

Set	FR	Metal salt solution		Supercritical water		Re	Ri	$v_{th}$ (m/s)	Yield (kg/h)
		Flow rate (g/min)	Velocity (m/s)	Flow rate (g/min)	Velocity (m/s)				
FS1	0.25	300	0.10	1200	1.86	59385	8.2	0.57	0.3
	0.5	600	0.20			77200	5.0	0.61	0.7
	0.75	900	0.30			87592	4.0	0.61	1.0
	1	1200	0.40			96500	3.5	0.64	1.3
	1.25	1500	0.50			102439	3.2	0.67	1.6
	1.5	1800	0.60			108377	2.9	0.70	1.7
FS2	0.25	600	0.20	2400	3.72	118763	32.8	1.15	0.7
	0.5	1200	0.40			154392	19.9	1.22	1.3
	0.75	1800	0.60			175176	16.2	1.24	2.0
	1	2400	0.79			192990	13.9	1.28	2.7
	1.25	3000	0.99			204866	12.8	1.34	3.1
	1.5	3600	1.19			216743	11.8	1.40	3.2
FS3	0.25	1200	0.40	4800	7.44	237507	131.2	2.28	1.3
	0.5	2400	0.79			308759	79.6	2.42	2.7
	0.75	3600	1.19			350323	64.7	2.45	4.0
	1	4800	1.59			385949	55.8	2.53	5.3
	1.25	6000	1.99			409699	51.1	2.65	5.9
	1.5	7200	2.38			433450	47.1	2.72	6.1

### Hydrodynamics and heat transport

The focus of the discussion in this subsection is the fluid flow, heat and species transport which play a significant role in the product formation in the infinity reactor. As noted earlier, 13 cross-sectional planes were created along the flow path at axial locations starting at 0.059 m till 1.57 m with equal separation of 0.126 m to facilitate post-processing. The distances are measured from the inlet as shown in the Fig. 2. These planes are named P1 through P13 in the figure. At these locations, the flow averaged velocity, density, temperature and concentration of the species were calculated and presented here.

**Figure 4:**
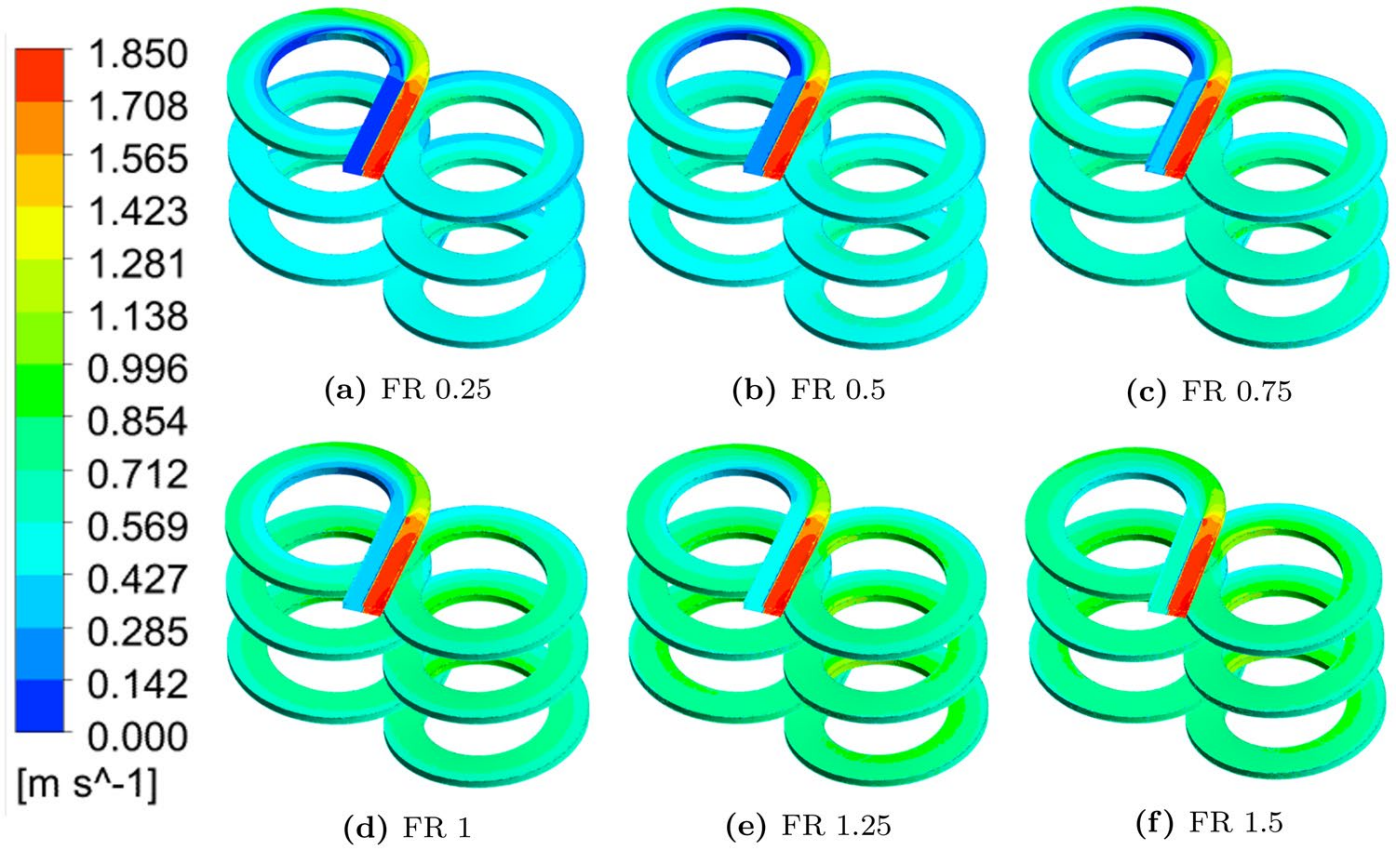
Contour plots of reaction mixture velocity with different flow ratios for the flow set FS1. Images used courtesy of ANSYS, Inc.

The contours of flow fields within the reactor channel carrying MS and SCW streams at various FR values for flow set FS1 are shown in Fig. 4. It can be observed from the contour plot that the magnitude of the inlet velocity of the metallic precursor solution is always lower than that of the supercritical water for all the FR values. As the FR increases, the fully developed velocity fields within the inlet channel carrying SCW is distorted near the exit region of the bisection wall due to the interpenetration of the heavier MS phase. The velocity fields in the MS channel are affected to a less extent for low FR values.

**Figure 5:**
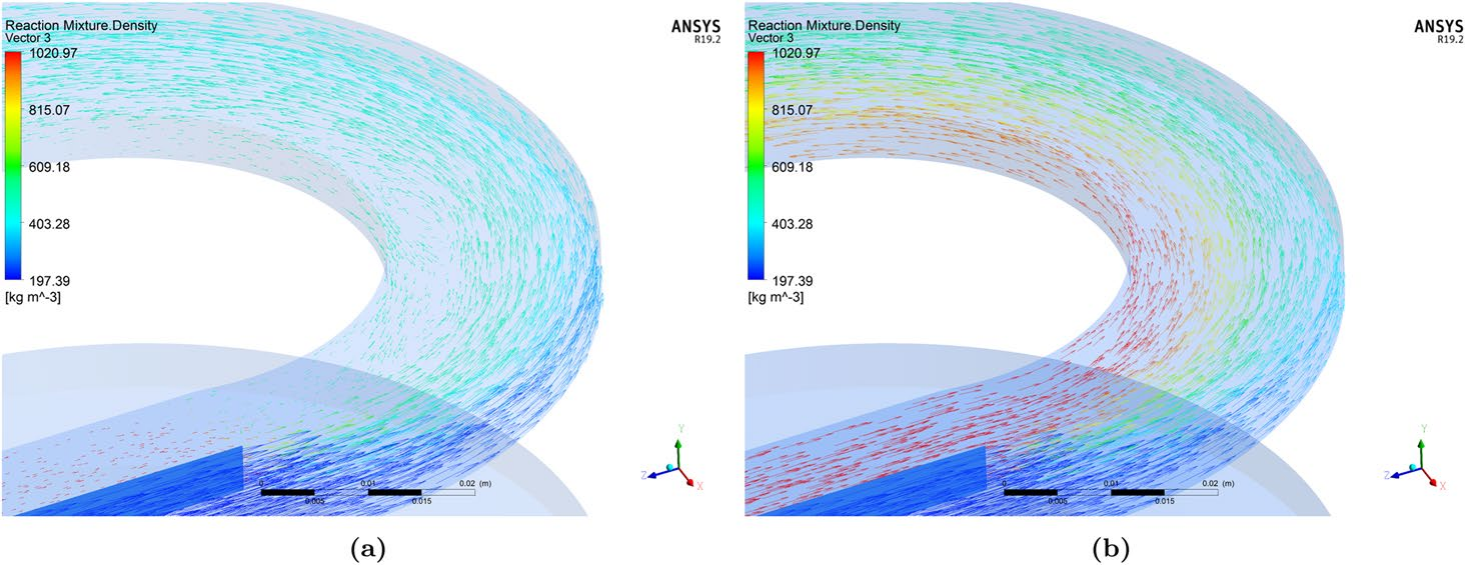
Velocity vector plot for FS1: (**a**) $FR=0.25$, and (**b**) $FR=1.5$. Images used courtesy of ANSYS, Inc.

In order to further understand the development of flow fields, the velocity vector plots corresponding to two extreme FR values for FS1 are shown in Fig. 5. In these plots, the length of the arrow signifies the magnitude of the velocity and the color indicates the fluid density. When the FR value is 0.25, the reverse flow of higher density stream leading to back-mixing can be observed in Fig. 5a in the immediate downstream of the inlet separator. The reader may have to zoom-in on the image to see the direction of the arrows clearly. This phenomenon is nearly absent when FR is 1.5, as can be seen in Fig. 5b. Here, a more gradual gradient in density and velocity fields are observed. It may be expected that the flow fields for the intermediate FR values would lie between the two extremes shown here.

The significant density difference between MS and SCW ($\rho _{ms}/\rho _{scw} >3.5$) trigger an interaction between inertial and buoyancy forces determining the interpenetration, recirculation and back-mixing of the flow streams. The dominant mechanism can be identified using the Richardson number (Ri), defined as ${\text {Ri}} ={\text {Gr}}/{\text {Re}}^{2}$, where Re and Gr are Reynolds and Grashoff numbers, respectively. In turn, these are defined as: ${\text {Re}}=\rho {{D}}_{{T}}U/\eta$ and ${\text {Gr}} = g\beta \delta T D^3_{T} \rho ^2/\eta ^2$, where $\rho$: fluid density, *U*: velocity of the fluid in the inlet section, $D_T$: the inner hydraulic diameter of the spiral, *g*: the acceleration due to gravity, $\beta$: the coefficient of thermal expansion, $\delta T$: the temperature difference between the supercritical water and metallic precursor solution.

When ${{\text {Ri}} >1}$, the convection is dominated by buoyancy, otherwise it is dominated by inertial forces^4^. The Re and Ri calculated for the flow mixture at the end of the bisection wall in the inlet section are given in the Table 2 for all the simulation conditions. It can be observed that ${\text {Ri}} > 1$ for the entire operating regime explored, indicating the flow is dominated by buoyancy. The denser MS solution displaces the SCW as they come in contact at the end of bisection wall of the inlet section. It results in the penetration of MS into SCW stream causing the movement of lighter SCW towards the inner region. This leads to a significant decrease in the velocity of SCW stream near and beyond the bisection wall. The degree of penetration depends on FR for a given feed temperature, pressure and SCW flow rate.

**Figure 6:**
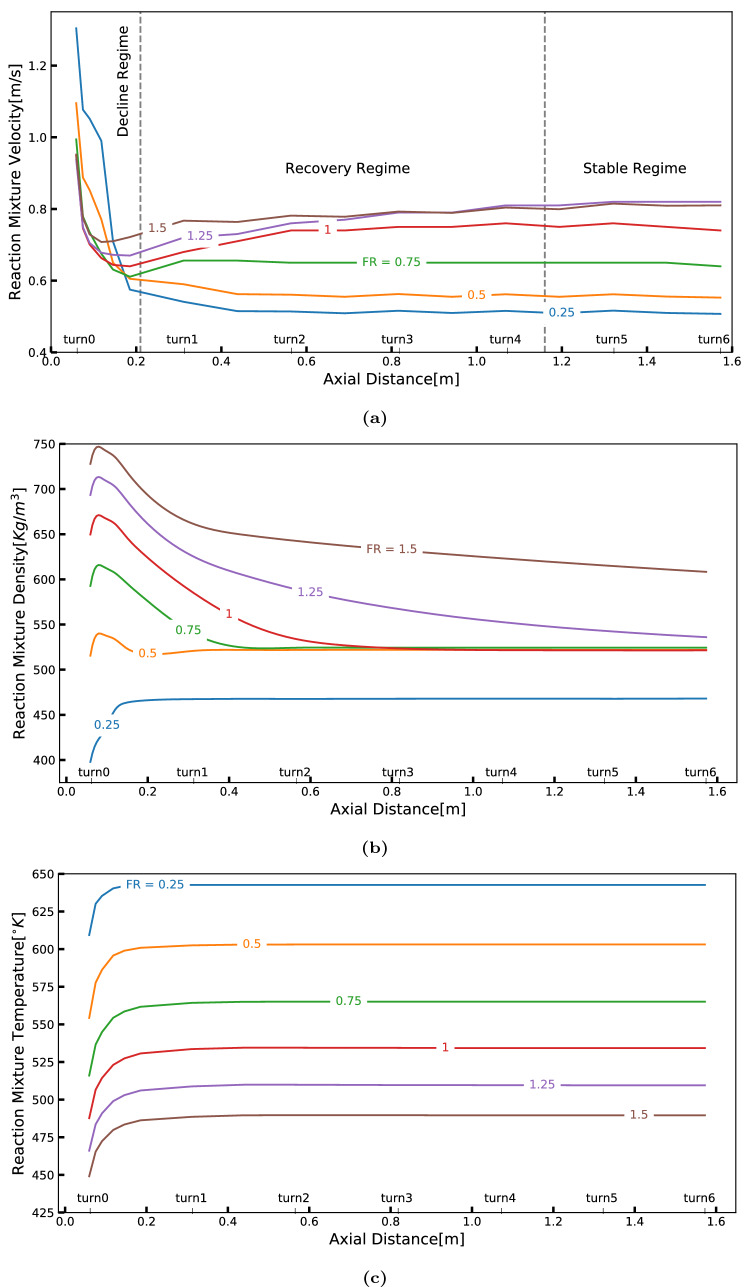
Axial profiles of selected mixture properties under different flow ratio: (a) mixture velocity, (**b**) mixture density, and (**c**) mixture temperature.

The flow behavior can be further elucidated by analyzing the profile of flow averaged velocity, density and temperature of the reaction mixture at different cross-sectional planes within the reactor shown in Fig. 6. For cases with $FR > 0.25$, the velocity profile showed three distinct regimes or regions named: declining, recovering and stabilizing regimes; the reasons for these names shall become obvious in the course of this study. For a better understanding, these are roughly marked in Fig. 6a. In the declining regime, the mixture velocity rapidly decreases to a threshold value within the first spiral turn due to the intense mixing caused by the interpenetration of metallic precursor solution into supercritical water stream. The convective transfer of heavier and cooler MS stream across the flow cross-section results in the density of the reaction mixture to raise and the temperature to drop as can be observed in Fig. 6b,c, respectively. Further, these phenomena cause an increased hold-up of the heavier MS stream slowing down the overall velocity. The threshold value of the reaction mixture velocity that marks the end of the decline-regime is termed as *threshold velocity* ($v_{th}$). It can also be viewed as a point of inflection of the axial velocity profile. It can be observed that the threshold velocity gradually increases with FR for a given flow set; this trend is also observed for other sets as shown in Fig. A.1 in the Supplementary material. The reader may find the density and temperature contour plots given in the supplementary material instructive (refer Figs. A.2 and A.3).

Beyond the threshold value, one can observe the velocity rising, in a regime that is referred to here as *recovery regime*. It is hypothesized that the convective mass transfer that dominated the decline regime is followed by active heat transfer between MS and SCW resulting in increasing temperature and decreasing density, thus accelerating the reaction mixture leading to the recovery regime. It can be observed from Fig. 6c that temperature equilibrates faster due to the combined effects of convective and radiative heat transfer. Ultimately, the velocity, density and temperature profiles reach steady values forming the *stable regime*.

Different velocity and density profiles are observed for the case of $FR=0.25$. These profiles may be explained by reviewing the velocity vector plots shown in Fig. 5a where a significant back-mixing is clearly seen. As the proportion of MS solution is sizably lower, back-mixing causes early homogenization of the reaction mixture with respect to composition and temperature. It aids in their faster equilibration resulting in a stable approach to uniform velocity, density and temperature profiles.

The counter intuitive observation of increasing temperature seen in Fig. 6c may be explained by the flow behavior of MS stream. This heavier and cooler fluid stream traveling at a lower velocity builds up (increased hold-up as mentioned earlier), and thus its contribution is stronger to the averaging process in the initial part of the reactor. This results in a temperature raise at the beginning of the reactor before stabilizing to a value between MS and SCW stream temperatures.

A simple linear regression model was obtained to capture the dependence of normalized threshold velocity on FR as given below:

14 $$\begin{aligned} \frac{v_{th}}{u_{SCW}} = 0.0542FR+0.2927 \end{aligned}$$

where $v_{th}$, and $u_{SCW}$ represent the threshold velocity, and velocity of SCW stream in the inlet channel, respectively. The correlation may be useful in estimating the threshold velocity for intermediate values of flow ratio (*FR*) and SCW stream velocity that is not simulated here.

### Chemical reactions

The hydrothermal reactions depend strongly on reaction mixture temperature and local precursor concentration that are in turn determined by the mixing characteristics of the reactor. As seen in the previous section, mixing of the two reactants in the infinity reactor is largely driven by their flow rates and density difference, assisted by the flow profile. Unique design of the reactor aids in rapid mixing of the reactants. The initial turns of the reactor act as a stirred tank or mixed flow reactor in ensuring that uniform concentration and temperature conditions are achieved with limited back-mixing. In most of the cases studied here, rapid evolution in temperature and composition is achieved within the first spiral turn of the reactor itself. Subsequent turns of the reactor help in improving the homogeneity to facilitate controlled nanoparticle growth. In this section, results on reaction progression are discussed and in the subsequent section, evolution of particle size distribution is analyzed.

**Figure 7:**
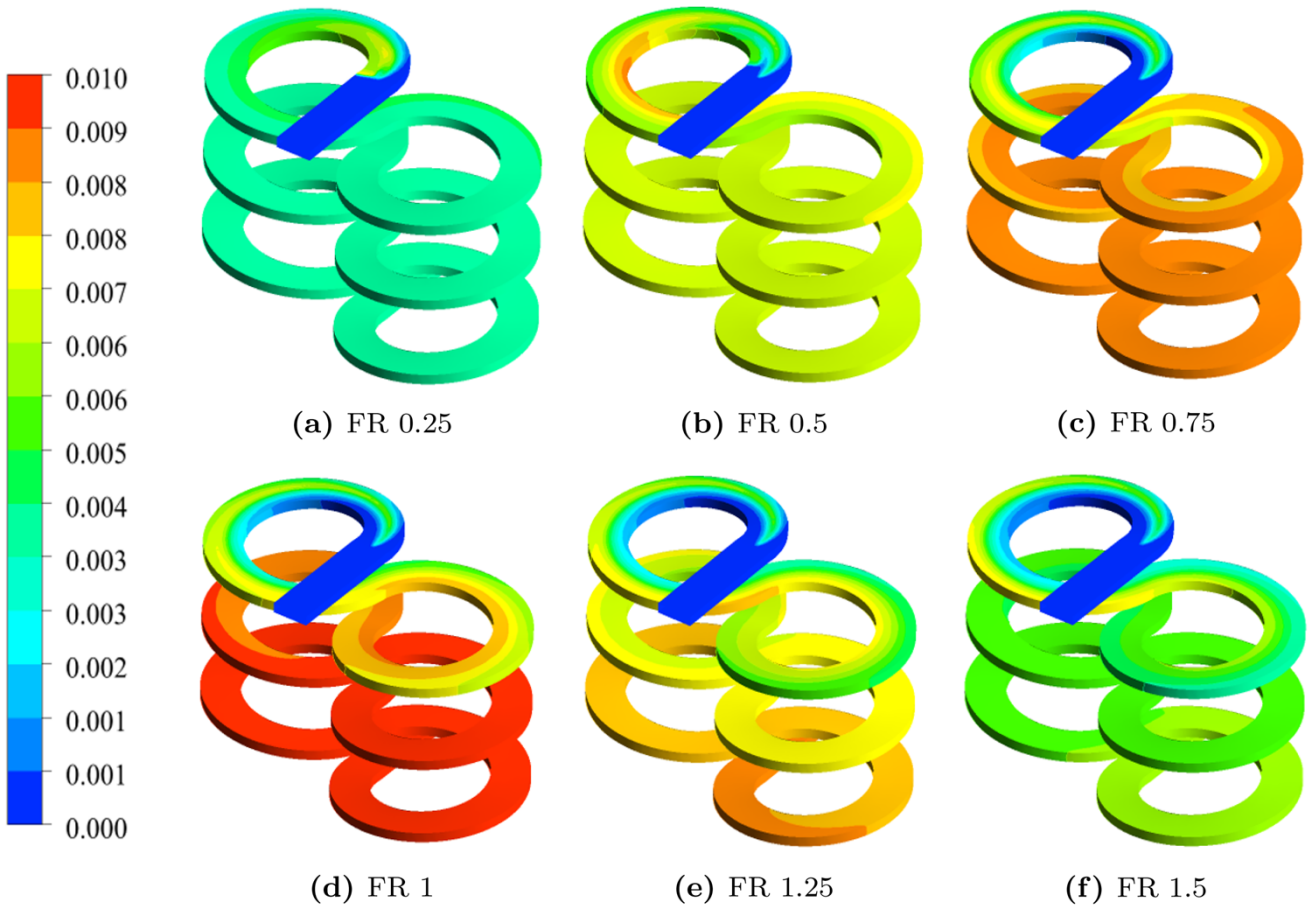
Contour plots of ceria mass fraction with different flow ratios for the flow set FS1. Images used courtesy of ANSYS, Inc.

Figure 7 depicts the contours of ceria produced along the flow path for all the FR values of flow set FS1. In the inlet section, as the streams remain separated due to the bisecting wall, it can be observed from the contour plots that the ceria concentration is zero (indicated in blue). Once the reactants start mixing, the reaction develops rapidly to form a new secondary phase of ceria and continues at various rates depending on the process flow conditions. Considering that the reactions are practically instantaneous under the explored temperature conditions, these plots also provide an effective means of visualizing how the reactant streams are mixing and form ceria. For $FR = 0.25$, it is observed that reaction went to completion in the first half of the turn itself. Moreover, the reaction is initially observed in the inner side of the spiral where the heavier MS solution is introduced before quickly spreading to the entire cross-section. This is due to the interpenetration of the MS stream into the SCW stream. As FR increases, the initial reaction front becomes sharper and gradually develops along the length of the reactor. It can be observed in Fig. 7 that exit ceria concentration increases with increasing FR up to 1.0, and then starts to decrease indicating the existence of optimum conditions, explored and discussed in detail as follows.

**Figure 8:**
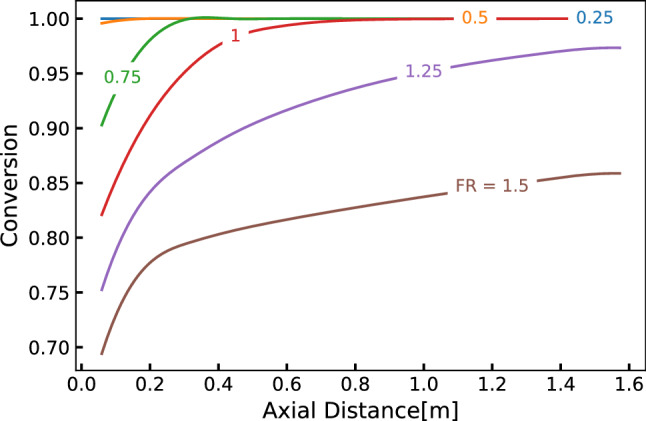
Fractional conversion of cerium nitrate to ceria for flow set FS1.

The axial profiles of conversion of the reactant cerium nitrate (A) are shown in Fig. 8 for flow set FS1. The mass conversion is defined as: $x_A = (M_{A0}-M_A)/M_{A0}$, where $M_{A0}$ and $M_{A}$ represents the initial mass concentration and mass concentration of cerium nitrate along the reactor, respectively. It can be observed that ceria conversion reaches 100 per cent at the beginning itself for low FR values ($FR \le 0.5$), whereas it rises gradually for higher FR values. In some of the cases, full conversion is not reached. This can be attributed to the drastic reduction in reaction mixture temperature at high FR values. For example, the reaction mixture temperature stabilizes at $600\,{\text {K}}$ for $FR=0.5$, whereas it reaches only $480\,{\text {K}}$ for $FR=1.5$. Although better mixing characteristics yield uniform concentrations of the precursor, lower reaction temperature and higher feed concentration limits the conversion. Since the hydrothermal reaction rate depends exponentially on the reaction temperature, as compared to its power law dependence on the concentration ($r \propto c^n$), the increase in FR beyond a point results in the reduction of reaction rate. Though not shown here, similar trends were observed for other flow sets.

**Figure 9:**
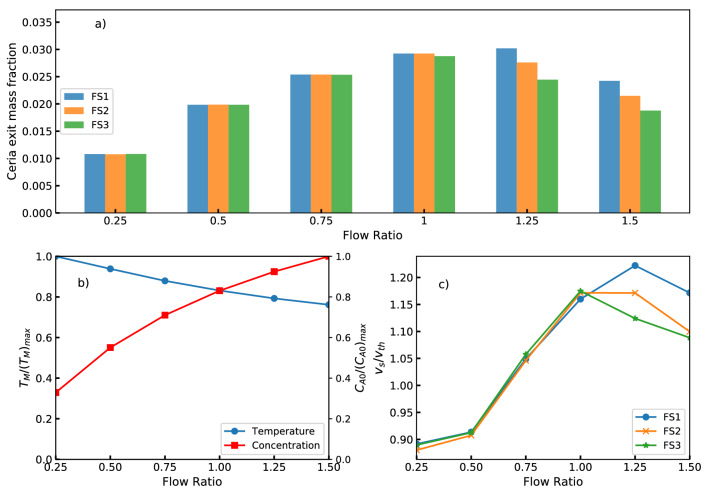
Consolidated view of key reaction parameters for all the flow sets and flow ratios: (**a**) comparison of ceria exit concentration, (**b**) normalized reaction mixture temperature and cerium nitrate concentration, and (**c**) scaled threshold velocity.

To understand these observations more comprehensively, the exit concentration of synthesized ceria nanoparticles for all the flow ratios and flow sets under the study are shown in Fig. 9a. All the reported values are flow-weighted average quantities computed at the outlet of the reactor. For a given flow set, it can be observed from the bar graph that the nanoparticle production increases with FR up to 1.0 and then shows a decreasing trend. The exit concentration is invariant when the SCW flow rate is doubled (FS2) or quadrupled (FS3), again till the FR of 1.0 and then shows a significant reduction in production of nanoparticles for FRs of 1.25 and 1.5. It is worth noting that the increasing trend continues till FR=1.25 for FS1 as an exception.

As observed earlier from Fig. 6c, the stabilized average reaction mixture temperature decreases with increasing FR. This is primarily due to the increasing proportion of MS solution for a fixed flow rate of supercritical water. Thus, increase in FR makes more of reactant or precursor available in the feed. The rate of reaction being a function of both reactant concentration and reaction temperature, there appears to be a trade-off between their effects in reaching the maximum ceria production. Figure 9b depicts the influence of FR on the effective reaction mixture temperature and the reactant concentration in the feed mixture. These variables are normalized with respect to their highest values among the explored conditions. Temperature was normalized with respect to $T_{max}$ of 642*K* observed for FR=0.25 and metallic salt mole fraction of $X_{A0}$ of 0.00147 for FR 1.5. As expected, when FR increases the effective reaction mixture temperature decreases and the concentration of the metallic salt increases. Interestingly, these two curves cross at FR=1. This could potentially represent the trade-off point discussed earlier where the effects of temperature and concentration on the rate of reaction combine to give the maximum overall ceria production rate.

It was hypothesized that $v_s/v_{th}$ that quantifies the relative velocity gain after reaching the threshold velocity is proportional to the intensity of active mixing in the reactor. This ratio may be called *velocity recovery ratio*. Its dependence on FR for various flow sets is shown in Fig. 9c. It can be observed from the figure that the overall flow characteristics inside the reactor remain unchanged for different flow sets till $FR\le 1$. The velocity recovery ratio decreases as FR increases beyond this range. For FS1 and $FR>1$, the ratio increases moderately. This decreasing trend is more pronounced for higher flow sets. These observations reinforce that this hypothesis deserves some merit.

### Particle characteristics

**Figure 10:**
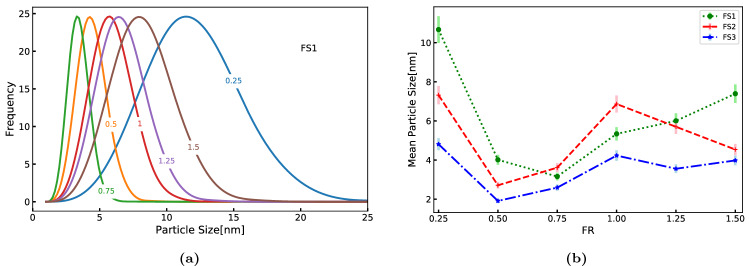
Characteristics of ceria PSDs predicted at the exit of the reactor: (**a**) PSD for different flow ratios in FS1, and (**b**) mean particle size and standard deviation of PSD (shown as bars around the mean) predicted at the exit.

The predicted Particle Size Distributions (PSD) and particle mean size at the exit of the reactor for different values of FR under flow set FS1 are shown in Fig. 10. For the lowest FR studied, $FR=0.25$, the PSD is relatively broad and mean diameter is high. However, the PSD becomes narrower with lower mean as FR increases up to 0.75 where the minimum mean size is observed for FS1. Further increase in FR causes the PSD to become wider with increasing mean size. This behavior can be attributed to the interplay among the rates of particle formation and growth events, such as nucleation, diffusional growth and coagulation. For flow sets FS2 and FS3, the minimum mean particle size is observed for $FR=0.5$. It is interesting to note that the range of mean particle size is reduced as the flow set changes from FS1 to FS3. This is primarily due to the drop in residence time for these sets, and for higher FR values it may further be compounded by the effect of reduced super-saturation.

In order to understand this further, average values of key process variables in the reactor and the corresponding average nucleation and coagulation rates at different FR values for FS1 are reported in Table A.1 (in supplementary material). As discussed in the previous subsection, when the FR increased, the reaction rate or formation of ceria decreased as temperature dropped. It resulted in the nucleation rate reaching a minimum for $FR=0.5$ and a continuous decline in the coagulation rate. This behavior is attributable to the fact that nucleation rate depends on both temperature and ceria concentration in a more complex manner whereas coagulation rate is directly proportional to temperature. Interestingly, the super-saturation continues to increase with FR. The interplay between nucleation, diffusional growth and coagulation rates determine the final PSD. Since nucleation and coagulation rates are high at $FR=0.25$, particle formation and growth were high and resulted in a wide PSD. However, for $FR=0.5$ and 0.75, nucleation and coagulation rates decreased, causing a drop in the number of particles leading to a narrow PSD with lower mean. The marginal increase in supersaturation and low temperatures appeared to have slowed down the particle growth process explaining the reduced mean size.

For $FR > 0.75$, nucleation rate increased, the coagulation rate continued to decrease and the increasing supersaturation aided the particle growth process despite the lower temperatures. The combined effect of these factors led to a broader PSD. These results suggest that lowering the FR to 0.75 is more favorable for achieving narrow PSD with a lower mean. It is worth recalling that the conversion is not affected significantly for values up to $FR=1$.

**Figure 11:**
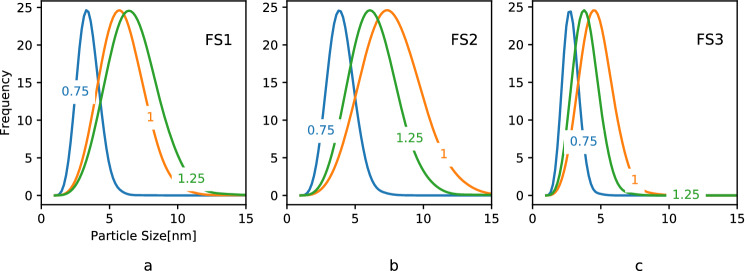
Particle size distributions at the exit of the reactor with selected flow ratios for all the flow sets: (**a**) FS1, (**b**) FS2, and (**c**) FS3.

The PSD for selected FR values for all the flow sets are shown in Fig. 11. It can be observed that the trends of PSD are similar in FS2 and FS3. In all the cases, $FR=0.75$ is favorable for achieving a narrower PSD with a lower mean size. However, the trends of PSD in FS2 and FS3 are different from FS1 as they showed double minima. This is also observed in Fig. 10b. It must be kept in mind that the total flow rate doubles in FS2 and quadruples in FS3 when compared to FS1 reducing the mean residence time by the same factor. Further, in these flow sets there appears to be a shift in nucleation dominant regime to particle growth dominant regime.

**Figure 12:**
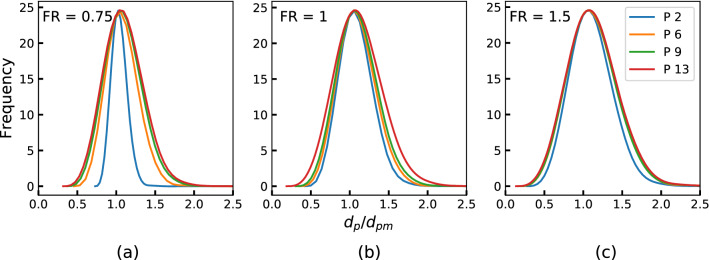
Development of normalized particle size distributions with different flow ratios for FS1 along the length of the reactor: (**a**) $FR=0.75$, (**b**) $FR=1.0$, and (**c**) $FR=1.5$.

**Figure 13:**
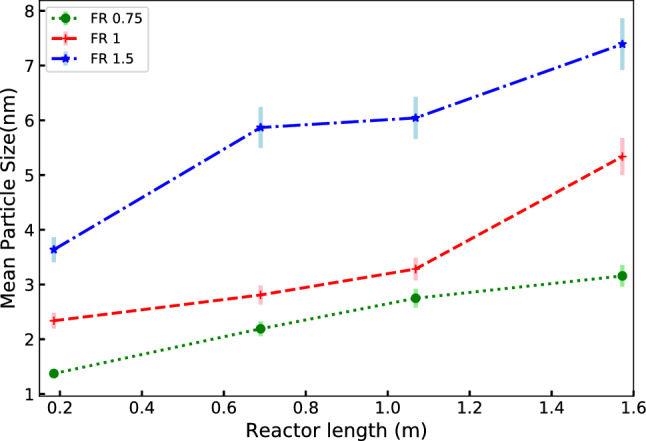
Growth of mean particle size for selected flow ratios with FS1 along the length of the reactor.

The PSDs in normalized particle size along the axial positions of the reactor are shown in Fig. 12. These results are often used to observe if the particle size distribution evolution has reached a stable growth phase with a single parameter (mean particle size) describing it. Under this condition, the mean size can be used in the design or control of the reactor. It can be observed that PSD reaches self-similar PSD for all the flow sets. The corresponding mean size as a function of the length of the reactor is shown in Fig. 13 for FS1. For runs with higher FR values the growth rates are higher. It may be safely projected that provisioning additional length of the reactor can help in increasing both the conversion and particle size if so desired.

It is worth comparing the infinity reactor with the T-mixer as both are continuous flow reactors with similar mean residence time. Based on the results presented here and the results from earlier works reported in the literature, it can be observed that smaller particles are produced in the infinity reactor compared to the T-mixer^11^. The mean size of particles produced in the infinity reactor varies in the range 1.9–10.7 nm whereas it is in the range of 44.9–63.7 nm in the T-mixer^11^. This can be attributed to the better mixing characteristics of the infinity reactor. The presence of significant back mixing in the T-mixer results in longer residence times at high temperature allowing the particles to grow to a bigger size^11,30^. Moreover, the throughput in the infinity reactor ranges from 10.5 to 63 g/min, which is nearly 10 times greater than the values reported for the T-mixer (1 to 6 g/min).

It is worth highlighting that while the article exclusively explored production of ceria nanoparticles through hydrothermal process, the infinity reactor and the methodology used are generic, and can be used to design, simulate, and optimize the production of other nanoparticles through processes that require rapid mixing of fluids of different densities.

## Conclusions

A novel infinity reactor was proposed for the scale-up of continuous nanoparticle production through hydrothermal method. The efficacy and feasibility of the reactor for continuous nanoparticle synthesis was validated using a coupled CFD-PBM. The mixing behavior, reaction kinetics, heat transfer and particle formation and growth within the reactor under different operating conditions have been studied. The fluid flow, temperature and density fields within the reactor showed three distinct regimes: declining, recovering and stable flow configurations. The extent of each of these regimes was observed to be a strong function of flow ratio between the two reactants.

The analysis of mixing and chemical reactions suggests that infinity reactor provides two different reaction environments: an initial mixed flow reactor followed by a plug flow reactor. The rapid mixing in the mixed flow zone ensures that thermal and concentration homogeneity is quickly achieved aiding the fast chemical reactions and the subsequent plug flow zone enables uniform conditions for the evolution of PSD. Thus, the infinity reactor was demonstrated to provide the required physico-chemical environment to produce nanoparticles with a tight control on the particle characteristics. The flow ratio was found to be a key factor in determining the yield and characteristics of the nanoparticles. Future work is needed and is being planned for exploring the production of nanoparticles in the infinity reactor experimentally.

## Supplementary Information

Supplementary Information.
