# Supplementary material for: A novel spiral infinity reactor for continuous hydrothermal synthesis of nanoparticles
Source: Sci Rep. 2022 May 21;12:8616. doi: 10.1038/s41598-022-11141-8 (PMC9124214; doi:10.1038/s41598-022-11141-8)
Supplement: Supplementary file 1 — Supplementary Information. [file 41598_2022_11141_MOESM1_ESM.pdf]

Appendices

A Supplementary Material

This section provides additional figures and tables that are referred in the main text but relegated to the supplementary to control the length of the article.

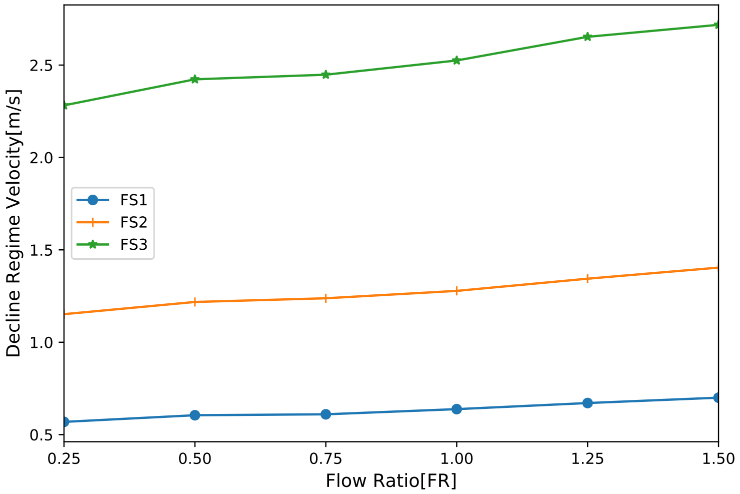

Figure A.1: Threshold velocity (v_{th}) as a function of flow ratio for all the flow sets

Table A.1: Comparison of averages of various factors that determine particle growth dynamics with different flow ratio for the flow set FS1

FR	Avg. Temp. (K)	Avg. Velocity (m/s)	Ceria conc. (mol/L)	Solubility (mol/kg)	Super sat.	Nucleation rate (#/m^3s)	Coagulation kernel (m^2/s)
0.25	639.32	0.6688	0.048398	5.00E-04	9.68E+01	3.66E+20	3.78E-16
0.5	596.88	0.6491	0.048398	4.00E-04	1.21E+02	8.46E+19	2.62E-16
0.75	558.08	0.6810	0.04059	2.00E-04	2.03E+02	8.84E+19	2.12E-16
1	527.48	0.7336	0.039666	9.00E-05	4.41E+02	3.47E+20	1.78E-16
1.25	503.19	0.7678	0.036027	4.50E-05	8.01E+02	6.08E+20	1.53E-16
1.5	483.61	0.7827	0.031561	2.10E-05	1.50E+03	1.24E+21	1.34E-16

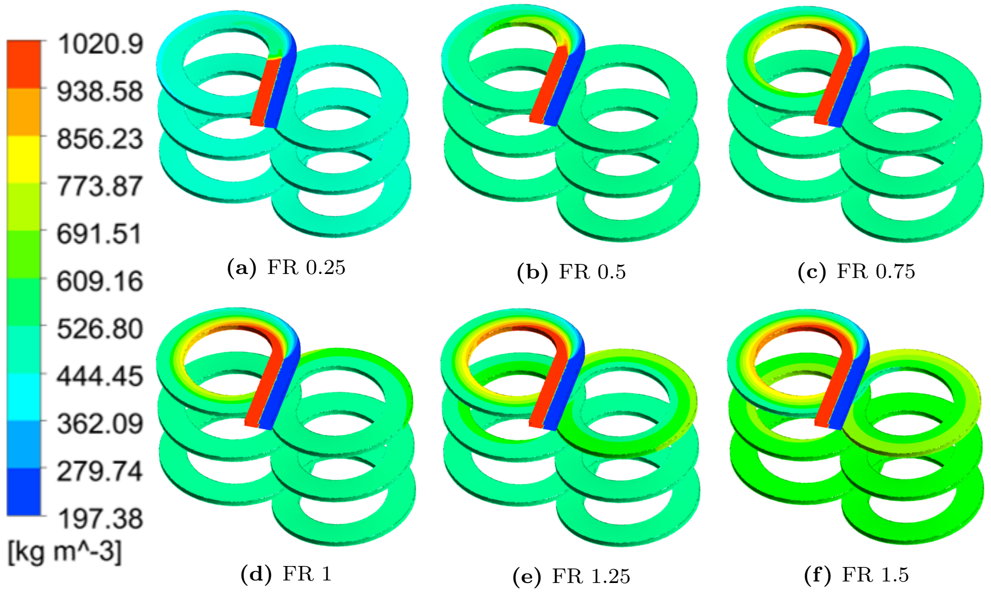

Figure A.2: Contour plots of reaction mixture density with different flow ratios for the flow set FS1. Images used courtesy of ANSYS, Inc.

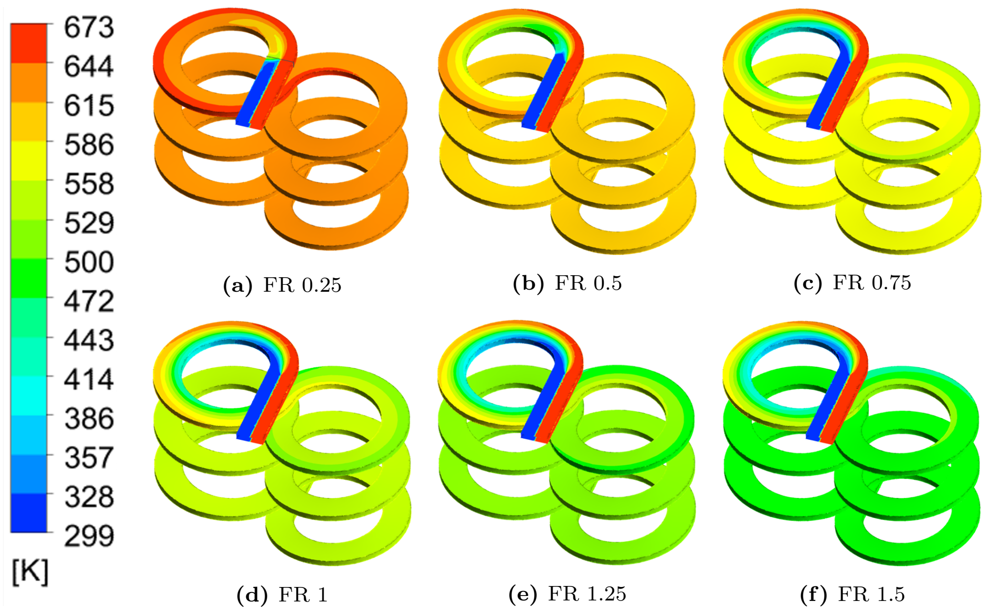

Figure A.3: Contour plots of reaction mixture temperature with different flow ratios for the flow set FS1. Images used courtesy of ANSYS, Inc.
